# Catestatin Induces Glucose Uptake and GLUT4 Trafficking in Adult Rat Cardiomyocytes

**DOI:** 10.1155/2018/2086109

**Published:** 2018-10-02

**Authors:** Maria Pia Gallo, Saveria Femminò, Susanna Antoniotti, Giulia Querio, Giuseppe Alloatti, Renzo Levi

**Affiliations:** ^1^Department of Life Sciences and Systems Biology, University of Turin, Via Accademia Albertina 13, 10123, Turin, Italy; ^2^Department of Clinical and Biological Sciences, University of Turin, Regione Gonzole 10, 10043, Orbassano (TO), Italy

## Abstract

Catestatin is a cationic and hydrophobic peptide derived from the enzymatic cleavage of the prohormone Chromogranin A. Initially identified as a potent endogenous nicotinic–cholinergic antagonist, Catestatin has recently been shown to act as a novel regulator of cardiac function and blood pressure and as a cardioprotective agent in both pre- and postconditioning through AKT-dependent mechanisms. The aim of this study is to investigate the potential role of Catestatin also on cardiac metabolism modulation, particularly on cardiomyocytes glucose uptake. Experiments were performed on isolated adult rat cardiomyocytes. Glucose uptake was assessed by fluorescent glucose incubation and confocal microscope analysis. Glut4 plasma membrane translocation was studied by immunofluorescence experiments and evaluation of the ratio peripheral vs internal Glut4 staining. Furthermore, we performed immunoblot experiments to investigate the involvement of the intracellular pathway AKT/AS160 in the Catestatin dependent Glut4 trafficking. Our results show that 10 nM Catestatin induces a significant increase in the fluorescent glucose uptake, comparable to that exerted by 100 nM Insulin. Moreover, Catestatin stimulates Glut4 translocation to plasma membrane and both AKT and AS160 phosphorylation. All these effects were inhibited by Wortmannin. On the whole, we show for the first time that Catestatin is able to modulate cardiac glucose metabolism, by inducing an increase in glucose uptake through Glut4 translocation to the plasma membrane and that this mechanism is mediated by the AKT/AS160 intracellular pathway.

## 1. Introduction

In the last decade, the 21-aa endogenous peptide Catestatin (Cts) has emerged as a multiple regulator of several physiological processes, including catecholamine release, baroreceptor activation, sympathetic nervous system activity, fat metabolism, antimicrobial activity, human neutrophil, monocyte, and mast cell migration and myocardial function [1]. In particular respect to the cardiovascular system Cts exerts cardiosuppressive effects both centrally and peripherally. Central effects are elicited by the improvement of baroreflex sensitivity and heart rate variability [2–4]. Peripheral effects consist in the decrease of blood pressure (stimulating histamine release [5] and evoking vasodilation [6]) and in the inhibition of catecholamine secretion [7]. Moreover, as Chromogranin A (CgA) is directly produced in the myocardium itself and processed to Cts [8, 9], Cts can exert direct influence on myocardial function. In particular in Langendorff-perfused rat heart Cts induces negative inotropic and lusitropic effects under basal and stimulated conditions (isoproterenol and endothelin), through ß2-AR-Gi/o-NO-cGMP dependent pathway [10]. In rat papillary muscles and isolated cardiomyocytes Cts exerts an antiadrenergic effect through a PI3K-AKT-eNOS mechanism [11]. On the whole these results suggest that Cts can protect the heart against sympathetic overstimulation.

Catestatin was also tested for its potential cardioprotective activity respect to ischemia/reperfusion damage. In isolated rat heart, Cts reduced the postischemic rise of diastolic LVP and improved postischemic recovery of developed LVP [12], through PI3K/AKT and PKC pathway [13]. In addition, in isolated adult rat cardiomyocytes subjected to simulated ischemia/reperfusion protocol, Cts induced an increase in cell survival with a mechanism mediated by AKT-GSK3ß-PLB-eNOS, converging on the preservation of mithocondrial membrane potential [14]. Moreoever, Cts showed also preconditioning effect, reducing the cardiac ischemia/reperfusion injury via binding to the cholinergic muscarinic type 2 receptor and via activation of the ERK1/2 and PI3K/AKT pathway, thus inhibiting the endoplasmatic reticulum stress-induced cellular apoptosis [15].

Based on these previous studies we pointed our attention on heart metabolism as a further potential target of this peptide, and in particular we focused on the improvement of glucose uptake, which represents a basic target for cardioprotective strategies. Cardiac glucose uptake can be impaired in several pathological conditions, due as an example to failure in insulin stimulation of Glut4; therefore the presence of an alternative pathway might be developed into a protective strategy.

## 2. Materials and Methods

### 2.1. Animal Care and Sacrifice

Experiments were performed on rats which were allowed* ad libitum* access to tap water and standard rodent diet. The animals received human care in compliance with the Guide for the Care and Use of Laboratory Animals published by the US National Institutes of Health (NIH Publication No. 85-23, revised 1996) and in accordance with Italian law (DL-116, Jan. 27, 1992). The scientific project was supervised and approved by the Italian Ministry of Health, Rome, and by the ethical committee of the University of Torino. Young adult rats were anaesthetized by i.p. injection of Pentobarbital (Nembutal, 100 mg/Kg) and killed by stunning and cervical dislocation.

### 2.2. Solutions and Drugs

Tyrode standard solution contained (mM): 154 NaCl, 4 KCl, 2 CaCl_2_, 1 MgCl_2_, 5.5 D-glucose, 5 HEPES, and pH adjusted to 7.34 with NaOH.

All chemicals were purchased from Sigma Aldrich unless otherwise specified.

### 2.3. Adult Rat Ventricular Cells Isolation

Isolated ventricular cells were obtained from adult rat hearts by enzymatic dissociation as described previously [11]. Briefly, after sacrifice, the rat hearts were explanted, washed in modified Ca^2+^-free Tyrode solution, and cannulated via the aorta. All the following operations were carried on under a laminar flow hood. The heart was perfused at a constant flow rate of 10 ml/min with Ca^2+^-free Tyrode solution with a peristaltic pump for approximately 5 min (37°C) to wash away the blood and then with 10 ml of Ca^2+^-free Tyrode supplemented with collagenase (0.3 mg/ml) and protease (0.02 mg/ml). Hearts were then perfused and enzymatically dissociated with 30 ml of Ca^2+^-free Tyrode containing 50 *μ*M CaCl_2_ and the same enzymatic concentration mentioned before. Atria and ventricles were then separated and the ventricles were cut in small pieces and shaken for 10 minutes in 20 ml of Ca^2+^-free Tyrode solution in presence of 50 *μ*M CaCl_2_, collagenase and protease.

### 2.4. Glucose Uptake Measurements

Cardiomyocytes plated on laminin-treated glass bottom dishes (MatTek Corporation) in Tyrode solution were stimulated for 15 minutes with 100 nM Insulin (Ins), 10 nM Cts (Phoenix Pharmaceuticals), or 10 nM Cts + 100 nM Wortmannin (Wm) and then loaded with 300 *μ*M 2-(7-Nitrobenz-2-oxa—1,3-diazol-4-yl)Amino)-2-Deoxyglucose) (2-NBDG, Life Technologies) for 20 minutes in the dark. 2-NBDG loading was performed in glucose-free Tyrode solution. After 1 wash in Tyrode standard solution cells were observed in confocal microscopy.

### 2.5. Immunofluorescence and Confocal Microscopy

Isolated cardiomyocytes plated on cover slide in Tyrode solution were stimulated for 15 minutes with 100 nM Ins, 10 nM Cts, or 10 nM Cts + 100 nM Wm. Cells were fixed for 30 minutes in 4% paraformaldehyde in 0.1 M phosphate buffer (PB), pH 7.3. After three washes with PBS, cells were incubated 20 minutes with 0.3% Triton and 1% bovine serum albumin (BSA) in PBS and stained with the primary antibodies 24h at 4°C. Cover slides were washed twice with PBS and incubated 1h at room temperature with the secondary antibody. After two washes in PBS cover slides were mounted on standard slides with DABCO and observed after 24h under confocal microscope. We used a polyclonal anti-Glut4 antibody (ThermoFisher Scientific, 1:100) and an Alexa Fluor 568 anti-rabbit (Molecular Probes, 1:1000).

Confocal fluorimetric measurements were performed using an Olympus Fluoview 200 laser scanning confocal system (Olympus America Inc., Melville, NY, USA) mounted on an inverted IX70 Olympus microscope, equipped with a 60X Uplan FI (NA 1.25) oil-immersion objectives. Image processing and analysis were performed with ImageJ software (Rasband, W.S., U. S. National Institutes of Health, Bethesda, MA, http:// rsb.info.nih.gov/ij/, 1997–2008).

### 2.6. Glut4 Translocation Analysis

Glut4 staining measurements of both cell periphery and cell interior were performed with the ABSnake plugin of the ImageJ Software. Briefly, for each cardiomyocyte the ABSnake plugin was employed to design a ROI (region of interest) band of 12 *μ*m around the plasma membrane and the fluorescence intensities of both the band and the cellular inside were evaluated.

### 2.7. Western Blot

Cardiomyocytes in suspension were stimulated for 15 minutes with 100 nM Ins, 10 nM Cts or 10 nM Cts + 100 nM Wm in Tyrode solution containing 50 *μ*M Ca^2+^, with gentle agitation. Proteins were extracted with Pierce RIPA buffer (Thermo Scientific) plus inhibitors cocktail. Proteins (50 *μ*g per lane) were resolved by 8% SDS–PAGE, transferred to a polyvinylidene fluoride membrane (PVDF, Millipore) in cold transfer buffer (25 mM Tris pH 8.3, 192 mM glycine, 0.1% SDS, and 20% methanol), and blocked overnight in TBST (10 mM Tris-HCl pH 7.5, 0.1 M NaCl, and 0.1% Tween 20) plus 5% BSA. PVDF membranes were incubated overnight at +4°C with primary antibody (AKT, 1:1000 dilution, Cell Signalling Technology; pAKT, 1:1000 dilution, Cell Signalling Technology; pAS160, 1:1000 dilution, Thermo Scientific; *α*-sarcomeric actin, 1:5000 dilution) in 1% BSA TBST, washed again and then incubated for 1h with the suitable horseradish peroxidase-conjugated secondary antibody (1:1500 goat a-rabbit, 1:10000 goat a-mouse, Thermo Scientific), followed by a second set of three washes in TBST. Bands were visualized with Western Lightning Plus ECL (Perkin Elmer). Protein levels were determined using ImageJ software and normalized corresponding to AKT level for pAKT and to *α*-actin level for pAS160 protein. Aspecific staining by secondary antibodies was checked.

### 2.8. Statistics

Data were expressed as mean ± standard errors. For differences between several mean values one-way ANOVA was performed. Differences with p<0.05 were regarded as statistically significant.

## 3. Results

### 3.1. Catestatin Increases Fluorescent Glucose Uptake

**Figure 1:**
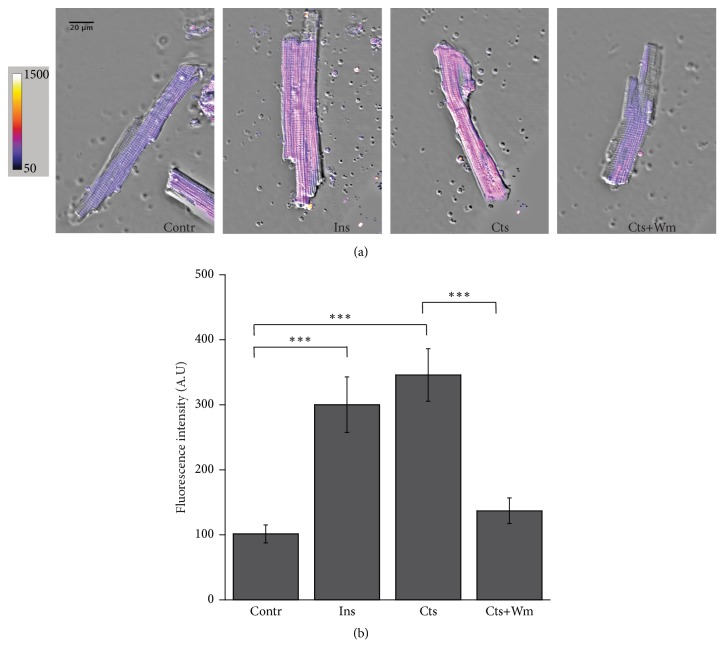
**Cts stimulates glucose uptake**. (a) Representative confocal images of adult rat cardiomyocytes incubated with the fluorescent glucose analog 2-NBDG in control, 100 nM Ins, 10 nM Cts, 10 nM Cts + 100 nM Wm. Pseudocolor images better show the fluorescence intensity variation. (b) Bar graph summarizing the experiments of fluorescent glucose uptake. The mean fluorescence intensity was 101.74±13.8 in control (n=72), 300.23±42.73 for Ins (n=30), 346.00±40.30 for Catestatin (n=52), and 137.12±19.63 for Cts+Wm (n=25). ^*∗∗∗*^p<0.001.

In order to investigate the potential role of Cts on glucose uptake, we performed measurements in confocal microscopy of the fluorescent glucose analog 2-NBDG absorption in adult rat cardiomyocytes. Cardiomyocytes were incubated with 300 *μ*M 2-NBDG for 20 minutes in the dark after 15 minutes stimulation with 10 nM Cts, 100 nM Ins (positive control), or 10 nM Cts + 100 nM Wm. Our results (Figure 1, panels (a) and (b)) showed a significant increase in intracellular fluorescence in Cts treated cells respect to control cells. The effect of Cts was comparable to that obtained with Ins and was reverted by Wm (Figure 1). The bar graph in Figure 1, panel (b), represents the mean fluorescence intensity in the different experimental conditions.

### 3.2. Catestatin Induces Glut4 Translocation from the Cytosol to the Plasma Membrane

**Figure 2:**
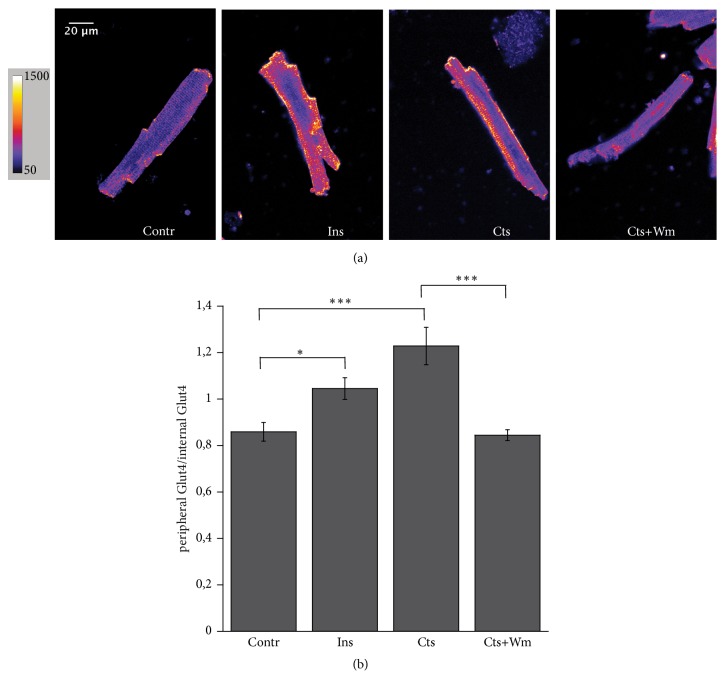
**Cts induces Glut4 translocation to the plasma membrane**. (a) Typical confocal images of Glut4 staining. Pseudocolor images better show the fluorescence intensity variation. (b) Bar graph summarizing the immunofluorescence experiments analysis. The peripheral vs internal Glut4 staining was 0.86±0.04 in control (n=21), 1.045±0.045 for Ins (n=18), 1.23±0.08 for Cts (n=16), and 0.84±0.02 for Cts+Wm (n=29). ^*∗*^p<0.05, ^*∗∗∗*^p<0.001.

To verify the involvement of Glut4 in the Cts dependent modulation of glucose absorption we performed immunofluorescence experiments using a Glut4 antibody, followed by a detailed image analysis of peripheral vs internal fluorescence staining, in the same experimental conditions described in Section 3.1. The results of these experiments are showed in Figure 2, panels (a) and (b): in control conditions Glut4 staining appears confined in the intracellular space, while after Cts and Ins stimulation the fluorescent signal is clearly localized to the peripheral plasmalemma, thus suggesting the Glut4 translocation. Treatment with Wm abolishes the effect of Cts.

### 3.3. Catestatin Stimulates AKT and AS160 Phosphorylation

**Figure 3:**
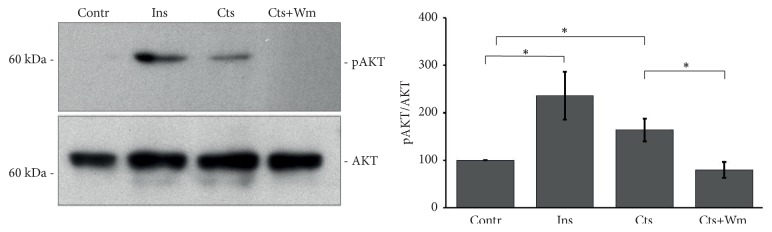
**Cts stimulates AKT phosphorylation**. Representative western blot experiment and summarizing bar graph showing AKT phosphorylation after Cts stimulation. Ins is the positive control. Wm reverted the effect of Cts; n= 4. Percentage values were as follows: Contr 100%, Ins 235.7±50.1%, Cts 163.8±24.2%, and Cts+Wm 79.9±16.8%. ^*∗*^p<0.05.

**Figure 4:**
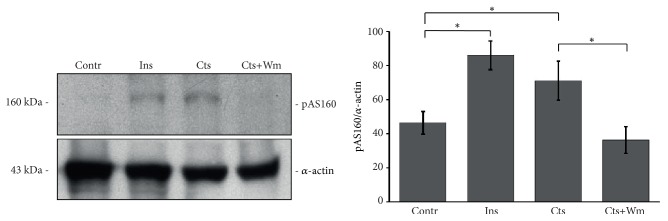
**Cts stimulates AS160 phosphorylation**. Representative western blot experiment and summarizing bar graph of AS160 phosphorylation induced by Cts. Ins is the positive control. Wm reverted the effect of Cts; n= 6. Percentage values were as follows: Contr 46.4±6.7%, Ins 85.9±8.5%, Cts 71.1±11.4%, and Cts+Wm 36.3±7.9%. ^*∗*^p<0.05.

As several studies demonstrated that AKT phosphorylation mediates multiple effects of Cts [4], we hypothesized that also the Cts-dependent glucose uptake was promoted by AKT and consequently by its target AS160. To verify this hypothesis we performed western blot experiments for AKT(Ser473) and AS160(Thr642) phosphorylation. As shown in Figures 3 and 4, Cts, similarly to Ins, stimulates both AKT and AS160 phosphorylation while Wm reverted this effect.

## 4. Discussion

In physiological conditions cardiomyocytes metabolism mainly takes place through fatty acids (70% of ATP production) and to a lesser extent (20% of ATP production) through glucose oxidation [16]. Significantly, the substrate preference of the heart switches throughout the life cycle as well as under physiological and pathological conditions. In particular, in response to ischemia or hypertrophy, mainly in the first phase of the diseases, the heart shifts towards glucose metabolism. This change ameliorates myocardial contractile efficiency by improving the stoichiometric ratio of ATP production to oxygen consumption and minimizes oxidative losses through mitochondrial respiratory chain uncoupling associated with fatty acid metabolism. On the contrary, the diabetic heart shows impaired glucose uptake and reduced glucose oxidation, associated with a shift towards fatty acid oxidation, intracellular storage of lipids, and cell death through apoptosis and autophagy [16, 17].

Grounded on these remarks, the search for agonists able to potentiate glucose metabolism and uptake could represent an important strategy to preserve heart function in disease conditions.

In this perspective we focused our attention on the vasoactive peptide Cts, extensively studied as a regulator of cardiac function and blood pressure and as a cardioprotective agent in both pre- and postconditioning through AKT-dependent mechanisms [4]. In particular, we hypothesized that in adult rat cardiomyocytes Cts could increase the first step in cardiac glucose metabolism and glucose uptake mediated by the Glut4 transporters, by inducing Glut4 translocation to the plasma membrane. We used 10 nM Catestatin, a mildly high concentration with respect to the plasmatic level measured in healthy subject [18].

As shown in Figures 1 and 2, Cts significantly enhanced both glucose uptake and Glut4 translocation.

Interestingly, these effects of Cts were inhibited by the PI3K inhibitor Wortmannin. Previously, we showed that Cts, both on endothelial cells and cardiomyocytes, activates the PI3K-AKT signaling, exerting indirect antiadrenergic and direct cardioprotective effects [11, 14]. The present results confirm the obligatory role of this pathway also in the modulation of glucose uptake. As it has been amply studied, the PI3K-AKT is also the main intracellular pathway used by insulin to induce glucose uptake [19].

Finally, we showed that Cts induces both AKT and AS160 phosphorylation (Figures 3 and 4). AS160 is a Rab GTP-ase activating protein that upon phosphorylation by AKT2 dissociates from Glut4 containing vesicles allowing Glut4 insertion in the plasma membrane. This mechanism in the heart has been also highlighted for Insulin, Neuregulin 1ß, Nefastin-1, and selective ß2-adrenergic receptor activation [19–22]. As shown in Figure 2, Cts stimulates both AKT and AS160 phosphorylation, suggesting that AKT and its substrate AS160 were responsible for the insertion of Glut4 in the plasma membrane.

## 5. Conclusions

In conclusion, we demonstrate for the first time a direct metabolic role of the vasoactive peptide Cts on cardiomyocytes, adding a new and important piece in the complex puzzle of its cardiovascular functions. Considering the paramount goals to improve heart metabolism in ischemia and hypertrophy and to point out agonists having an insulin-like effect in the diabetic heart, this study acquires remarkable value in the cardiovascular research field.

## Data Availability

The data used to support the findings of this study are available from the corresponding author upon request.
